# Optimization of Calcium Fluoride Crystallization Process for Treatment of High-Concentration Fluoride-Containing Semiconductor Industry Wastewater

**DOI:** 10.3390/ijms25073960

**Published:** 2024-04-02

**Authors:** Arindam Sinharoy, Ga-Young Lee, Chong-Min Chung

**Affiliations:** Department of Environmental Science & Biotechnology, Jeonju University, Jeonju 55069, Republic of Korea; arindam.sinharoy004@gmail.com (A.S.); dlrkdud1007@jj.ac.kr (G.-Y.L.)

**Keywords:** calcium fluoride, crystallization, fluidized bed reactor, semiconductor wastewater, response surface methodology

## Abstract

This study utilized a fluidized bed reactor (FBR) for fluoride removal from high-concentration fluoride-ion-containing simulated semiconductor industry wastewater and recovered high-purity CaF_2_ crystals. The effects of hydraulic retention time (HRT), pH, Ca^2+^ to F^−^ ratio, upflow velocity, seed size and seed bed height were investigated by performing lab-scale batch experiments. Considering fluoride removal and CaF_2_ crystallization efficiency, 5 h HRT, pH 6, seed height of 50 cm and [Ca^2+^]/[F^−^] ratio of 0.55 (mol/mol) were found to be optimum. The effect of the interaction between the important process parameters on fluoride removal was further analyzed using response surface methodology (RSM) experimental design. The results showed that all the individual parameters have a significant impact (*p* = 0.0001) on fluoride removal. SEM-EDX and FTIR analysis showed the composition of the crystals formed inside FBR. HR-XRD analysis confirmed that the crystalline structure of samples was mainly CaF_2_. The results clearly demonstrated the feasibility of silica seed material containing FBR for efficient removal and recovery of fluoride as high-purity calcium fluoride crystals.

## 1. Introduction

Fluoride ions (F^−^) are widely used in various industries, including semiconductor manufacturing, electronics, glass production, steelmaking and LCD fabrication [1]. However, the discharge of high-concentration fluoride in wastewater from these industries can pose significant environmental and public health concerns [2]. Fluoride is an essential element in the human body, but when present in concentrations exceeding 5 mg/L, it can lead to severe toxicity, potentially causing health issues such as osteoporosis, arthritis and neurological damage [3,4,5]. Consequently, the treatment of fluoride ions from industrial wastewater has emerged as a pressing environmental challenge.

Prominent sources of high-concentration fluoride-containing wastewater include the electronics industry (ranging from 300 to 4500 mg/L), semiconductor and display manufacturing (ranging from 400 to 1200 mg/L), and steel production (ranging from 6500 to 130,000 mg/L), with fluoride concentrations varying based on manufacturing processes [6,7,8]. Semiconductor industry wastewater is of particular concern, not only because of its high fluoride content but also because of the anticipated increase in its release to the environment in the future because of the rapid growth of the semiconductor industry worldwide [9,10].

Research on fluoride ion removal has been ongoing for the past few decades; the involved methods include coagulation/precipitation [1,11], adsorption [12], ion exchange [13], membrane processes [6] and electrocoagulation [14]. Among these, the most commonly utilized method is coagulation/precipitation with lime (Ca(OH)_2_) with the formation of calcium fluoride (CaF_2_) precipitates [15]. This approach offers advantages such as the ability to treat large volumes of wastewater in a short residence time and ease of operation. However, due to the low solubility of lime, its reactivity diminishes over time. Additionally, impurities present in wastewater can interfere with CaF_2_ precipitation, leading to reduced treatment efficiency [7]. Furthermore, coagulation/precipitation with lime generates inorganic sludge that is challenging to recycle due to its high content of impurities, including coagulants and polymers from the feedwater. While some of this inorganic sludge is currently repurposed as cement raw material, limited cement plant capacity for disposal raises concerns about potential secondary soil pollution from landfill disposal of excess sludge [15,16].

Therefore, there is a pressing need to develop technologies that not only treat fluoride ions from high-fluoride-containing wastewater but also facilitate the recovery of fluoride ions as valuable resources. According to the existing literature, calcium fluoride (CaF_2_) precipitation methods for the removal and recovery of fluoride ions as valuable resources can be one such useful process [17]. This technology is known to have the potential to replace conventional chemical coagulation/precipitation methods, producing minimal waste and generating high-purity fluoride calcium crystals [18,19,20]. Despite having such large potential, there are only a handful of studies on calcium fluoride crystallization mechanisms to treat fluoride-containing wastewater, and there are fewer studies on the effect of various process parameters. Hence, this study addresses the limited research in the field of fluoride-ion-containing wastewater treatment using calcium fluoride crystallization and aims to explore the effect of key process parameters such as hydraulic retention time (HRT), pH, upflow velocity, seed size and seed bed height on the treatment efficiency. The experiments employ a fluidized bed reactor (FBR) and a reactor well-suited for creating crystals with low moisture content and requiring minimal space for sludge treatment. The fundamental principle of the FBR reactor involves filling it with seed crystals, followed by the introduction of fluoride-containing wastewater in an upward flow to facilitate the adsorption of fluoride ions from the wastewater onto the seeds and subsequent CaF_2_ crystal formation.

## 2. Results and Discussion

### 2.1. Effect of Process Parameters

#### 2.1.1. Effect of Hydraulic Retention Time

**Figure 1:**
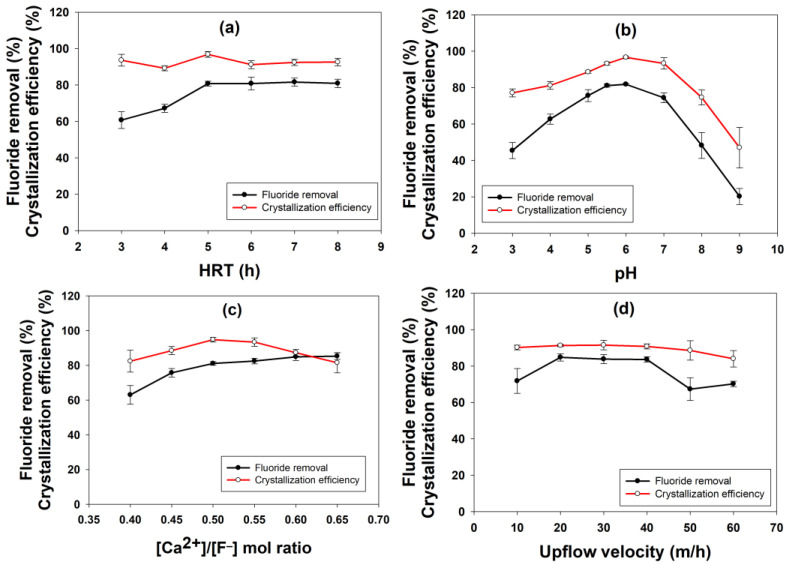
Effect of (**a**) HRT, (**b**) pH, (**c**) calcium to fluoride ratio and (**d**) upflow velocity on fluoride removal and calcium fluoride crystallization efficiency.

Figure 1a shows the effect of different HRTs on fluoride removal and crystallization efficiency. The fluoride removal was low for the initial two HRT values of 3 and 4 h; however, it increased at high HRT values (>5 h) and remained consistent (≈80%). The increase in HRT showed a positive impact on fluoride removal because it increased the contact time between the reactants, allowing for more fluoride to be adsorbed onto the seed particles [21]. Similarly, earlier observations were also made by other authors [22,23,24], although the optimum HRT value varied widely depending on the seed material used and other experimental conditions. The reason for stable removal efficiencies for other HRT values (6–8 h) must be due to the saturation of the active adsorption site onto the seed materials, which stagnated any further F^−^ ion removal [21]. The crystallization efficiency was more or less similar (>90%) for the six HRT values applied in this study, except for the 4 h HRT. Hence, based on these observations, 5 h was chosen as the optimum HRT for further experiments.

#### 2.1.2. Effect of pH

The change in pH showed significant variation in both fluoride removal and CaF_2_ crystallization efficiencies (Figure 1b). The optimum pH was determined to be 6, at which the fluoride removal and crystallization efficiencies were maximum (81.8% and 96.6%, respectively). The efficiencies declined at both lower and higher pH values than pH 6. The reason behind this could be that at low pH conditions, fluoride ions stabilize as hydrogen fluoride (HF) rather than calcium fluoride due to the excess amount of H^+^ ions produced at low pH conditions (Equation (3)) [25]. In contrast, the reverse reaction (in Equation (1)) to free fluoride ions is more favorable at pH greater than 5, which promotes the formation of CaF_2_ by combining F^−^ ions with Ca^2+^ ions available in the reactor (Equations (2) and (3)). Due to this reason, the fluoride removal and CaF_2_ crystallization efficiency improved with the increase in pH. The reduction in removal efficiencies at higher pH could be explained by the fact that OH^−^ ions produced at such conditions must have competed with the fluoride ions, resulting in the formation of unwanted products such as calcium hydroxide and calcium carbonate rather than calcium fluoride [26,27].

H^+^ + F^−^ ⟷ HF (1)

Ca^2+^ + 2Cl^−^ ⟷ CaCl_2_ (2)

Ca^2+^ + 2F^−^ ⟷ CaF_2_ (3)

#### 2.1.3. Effect of Ca^2+^ to F^−^ Ratio

The effect of calcium to fluoride ion molar ratio on the fluoride removal and CaF_2_ crystallization efficiency is depicted in Figure 1c. The fluoride removal efficiency gradually improved with an increase in the Ca^2+^ to F^−^ ratio, and maximum removal of 85% was obtained at a ratio of 0.6. Furthermore, an increase in the Ca^2+^ to F^−^ ratio to 0.65 did not show any significant improvement, and the fluoride removal efficiency remained almost the same as the value obtained previously for a ratio of 0.6. In the case of crystallization efficiency, the values varied between 82.4 and 94.8% for different Ca^2+^ to F^−^ molar ratios. However, the highest crystallization efficiency of 94.8% was obtained with a Ca^2+^ to F^−^ ratio of 0.5. The theoretical optimum Ca^2+^ to F^−^ ratio is 0.5 (Equation (5)), i.e., twice the amount of fluoride required for each mol of calcium to synthesize one mole of calcium fluoride. The experimental optimum ratios obtained in this study were slightly higher than the theoretical value, which shows that some amount of calcium must have been lost during the experiment to react either with other cations present in the wastewater or within the experimental setup. Similar better fluoride removal at a high Ca^2+^ to F^−^ ratio has been reported in the literature, where a 0.6–1.0 ratio showed the best results [17].

#### 2.1.4. Effect of Upflow Velocity

The effect of different upflow velocities on fluoride removal and crystallization efficiency is shown in Figure 1d. The fluoride removal efficiency increased from 71.8% to 84.8%, with an increase in the upflow velocity from 10 to 20 m/h. After this, the removal efficiency remained almost the same (83.8 and 83.6%) for the next two increases in upflow velocities to 30 and 40 m/h, respectively. However, further increase in upflow velocity to 50 and 60 m/h resulted in a drastic reduction in this value to 67.3% and 70.2%, respectively. The crystallization efficiency was consistently high for all the different upflow velocities applied to the reactor. The maximum crystallization efficiency obtained was nearly 91% at 20 to 40 m/h upflow velocities. However, the crystallization efficiency reduced gradually to below 90% with a further increase in the upflow velocity beyond 40 m/h. This indicates that neither a low nor a very high upflow velocity is suitable for fluoride removal using the crystallization process.

The bed expansion in the FBR-type system is directly proportional to the upflow velocity to the reactor and is responsible for the proper mixing of the reactant and their interaction with the seed materials [28]. Due to this reason, the desired level of mixing could not be achieved inside the FBR at low upflow velocity, which resulted in low fluoride removal at 10 m/h upflow velocity. Another point to be noted is that at high upflow velocity, due to higher fluidization, the smaller-sized particles are often expelled out from the reactor along with effluent resulting in poor removal performance as well as crystallization [27,28].

#### 2.1.5. Effect of Seed Bed Height

**Figure 2:**
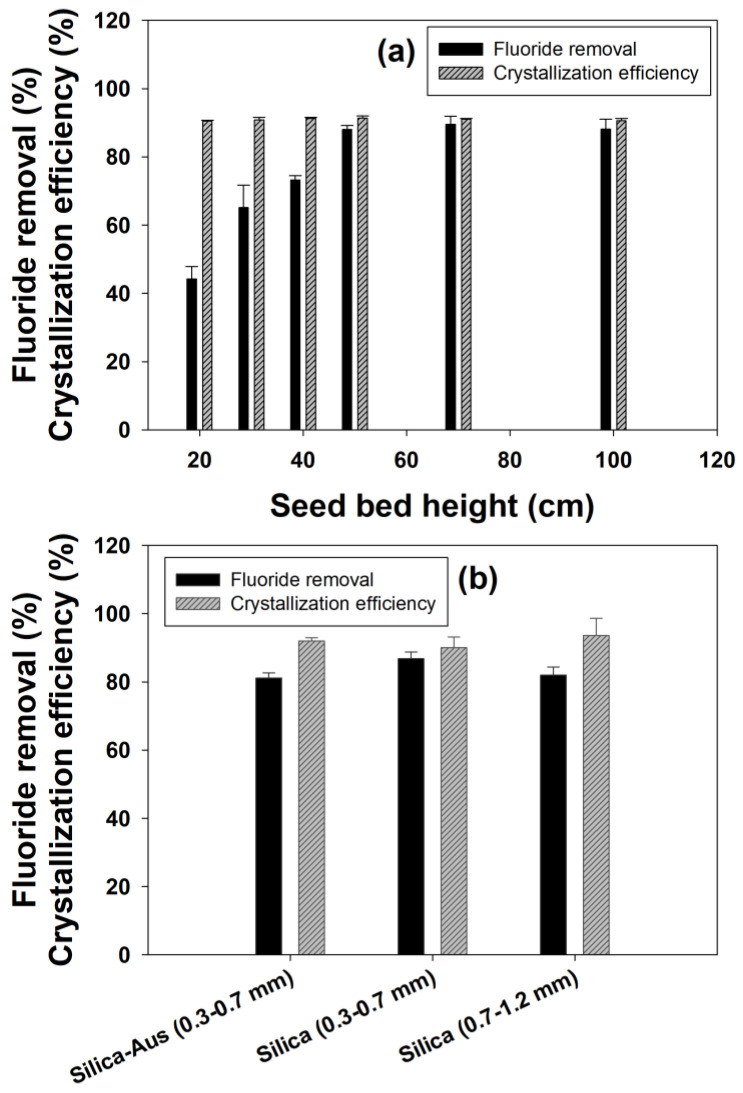
Effect of (**a**) seed bed height and (**b**) seed size on fluoride removal and calcium fluoride crystallization efficiency.

Figure 2a shows the effect of different seed bed heights on fluoride removal. The seed bed height is directly proportional to the number of seeds available inside the reactor for crystallization. The results showed that increasing the seed height had a positive impact on the fluoride removal, as with each stepwise increase from 20, seed bed height resulted in an increase in the removal efficiency. However, there was no further increase in the removal efficiency beyond 50 cm bed height, and the fluoride removal remained constant at nearly 88% for 50, 70 and 100 cm seed bed height. The crystallization efficiency remained near about 91% for all the six seed bed heights used in this study.

The increase in bed height increased the amount of seed present inside the reactor and consequently improved the fluoride removal efficiency. Furthermore, the induction period also reduces with an increase in seed bed height and seed amount, which in turn improves the crystallization efficiency. However, there was no improvement beyond 50 cm seed bed height, which could be explained by typical flow behavior in FBR [19]. In the case of an excess amount of seed in FBR, the movement of seed crystals became irregular to some degree, and despite having a high flow rate to maintain the same upflow velocity, the mixing is improper to keep all the seed suspended [19]. This failure in proper mixing causes a reduction in surface contact between the reactants and seed, negatively affecting the fluoride removal efficiencies.

#### 2.1.6. Effect of Seed Type

The effect of seed size on fluoride removal is shown in Figure 2b. Although it appears that the removal efficiencies were nearly similar for all the different particles, from the final results, it could be established that smaller-sized particles showed better fluoride removal efficiencies. Moreover, higher removal efficiencies (86.8%) were obtained with smaller (0.3–0.7 mm) sized silica sand out of three different types of particles used as seed material. This effect of seed size on fluoride removal is related to the higher specific surface area available for smaller-sized seed particles, which have an increased potential for reacting with fluoride and calcium [29]. The mechanism of fluoride removal depends on the initial adsorption of fluoride onto the seed surface, and it can improve greatly for small-size adsorbent particles due to this reason [30]. When comparing silica with other compounds as seed materials for fluoride removal, it is found that silica has better mixing capacity inside FBR due to its random movement in the liquid phase. This helps in better contact of the seed particles with the reactants (Ca^2+^ and F^−^), which improves their removal and crystallization efficiency [19].

#### 2.1.7. Effect of Initial Fluoride Concentration

**Figure 3:**
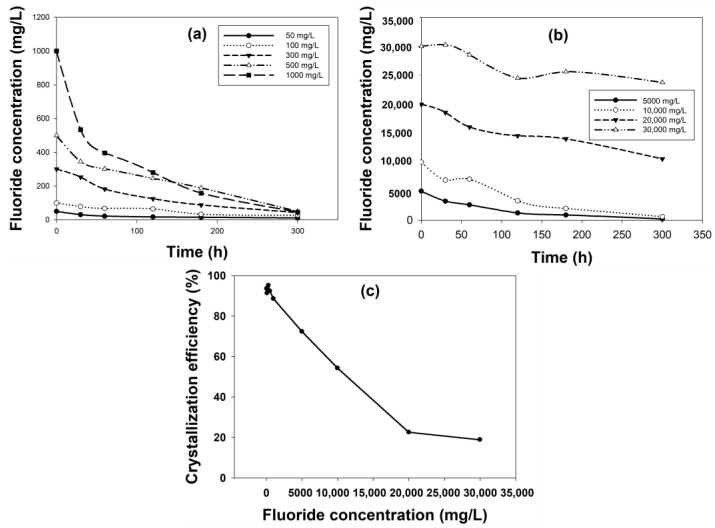
Effect of influent fluoride concentration on fluoride removal and crystallization efficiency: fluoride removal for initial fluoride concentration of (**a**) 50–1000 mg/L and (**b**) 5000–30,000 mg/L; (**c**) respective crystallization efficiency for all the initial fluoride concentrations.

The variation in influent fluoride concentration on fluoride removal by fluidized bed crystallization was studied and reported in Figure 3. The results showed that initial fluoride concentration had a great impact on its removal even though the [Ca^2+^]/[F^−^] ratio was kept the same. The fluoride removal for the two lowest initial fluoride concentrations of 50 and 100 mg/L were 78% and 72%, respectively (Figure 3a). The reason could be that at a low concentration, the amount of fluoride was not sufficient to react with calcium to form calcium fluoride crystals. Fluoride removal increased to 85.6% and 89.9%, with an increase in initial F^−^ concertation to 300 and 500 mg/L. The fluoride removal showed a further increase to ≈95% for 1000, 5000 and 10,000 mg/L initial F^−^ concentrations (Figure 3b). However, fluoride removal drastically depleted to 47.2% and 20.8% for the remaining two high initial fluoride concentrations of 20,000 and 30,000 mg/L, respectively.

The corresponding crystallization efficiency also varied depending on the initial fluoride concentration. The crystallization efficiencies were >90% for 50–500 mg/L, which went to 88.5% and 77.3% for 1000 and 5000 mg/L, respectively (Figure 3c). Further increase in influent fluoride concentration resulted in a drastic reduction in crystallization efficiency to 54% (at 10,000 mg/L F^−^ concentration) and later to ≈20% (at 20,000 and 30,000 mg/L of F^−^ concentration). The best performance for both the parameters was obtained for moderate to slightly high initial fluoride concentrations (300–10,000 mg/L), which showed that at these concentrations, the reactivity with calcium was maximum. When the initial fluoride concentration was very high to match the calcium-to-fluoride ratio, the calcium concentration was also very high (21,000 and 31,500 mg/L), at which condition the solubility of calcium reduced, and it formed a viscous solution resulting in scaling around the inner walls of the reactor in addition to precipitate formation. This could have caused poor removal performance at high initial fluoride concentration. This experiment was relevant to see the feasibility of this process in treating fluoride-containing wastewater from different industries with various concentrations. The results confirmed that an influent fluoride concentration of 10,000 mg/L or less is recommended for treatment using this process.

### 2.2. Optimization of Process Parameters Using RSM

Optimization of important process parameters for fluoride removal using the crystallization process was carried out using RSM. The experimental design provided 17 experimental runs with different combinations of the variables. The response to each of the experimental runs in the RSM design is provided in Table 1. A high calcium concentration (598 mg/L), high seed bed height (100 cm) and a moderate upflow velocity (35 m/h) resulted in the best fluoride removal (94.8%) in the experimental design. The effect of upflow velocity (A), calcium concentration (B) and seed bed height (C) on fluoride removal efficiency (Y) is depicted using the following second-order polynomial equation (Equation (4)) obtained from the regression analysis of the experimental data:

Y = 85.20 − 5.42A + 8.19B + 20.44C − 0.7000AB − 1.50AC + 1.43BC − 7.89A^2^ − 0.6625B^2^ − 21.01C^2^ (4)

where Y is the fluoride removal (%); A, B and C are the upflow velocity (m/h), calcium concentration (mg/L) and seed bed height (cm), respectively; AB, AC and BC are the interaction effects between these parameters.

The equation in terms of actual factors that can be used to make predictions about the response (fluoride removal) for given levels of each factor is provided herein (Equation (5)):

Fluorine removal = −44.96138 + 0.874000 upflow velocity + 0.109522 calcium conc. + 1.98975 seed height − 0.000243 (upflow velocity × calcium conc.) − 0.001500 (upflow velocity × seed height) + 0.000310 (calcium conc. × seed height) − 0.012620 upflow velocity^2^ − 0.000050 calcium conc.^2^ − 0.013133 seed height^2^ (5)

Here, the levels should be specified in the original units for each factor. The predicted values for different experimental conditions showed a high degree of similarity with the experimental results for fluoride removal (Table 1).

The statistical analysis of the results obtained from the experimental design was performed using analysis of variance (ANOVA), as shown in Table 2. A low *p*-value and a high F-value indicate the significance of the model and the individual parameters. From the ANOVA results, it is clear that the model is significant and can accurately predict the response from the experimental design. The predicted and adjusted coefficient of determination (R^2^) values are in reasonable agreement (<0.2 difference) with each other, showing a good fit of the data with the model. In this study, all the variables, as well as squared values of two variables (upflow velocity and seed bed height), showed a significant (*p* < 0.05) impact on fluoride removal. However, the interaction effects between the variables were insignificant (*p* > 0.05) within the range applied in this study.

**Figure 4:**
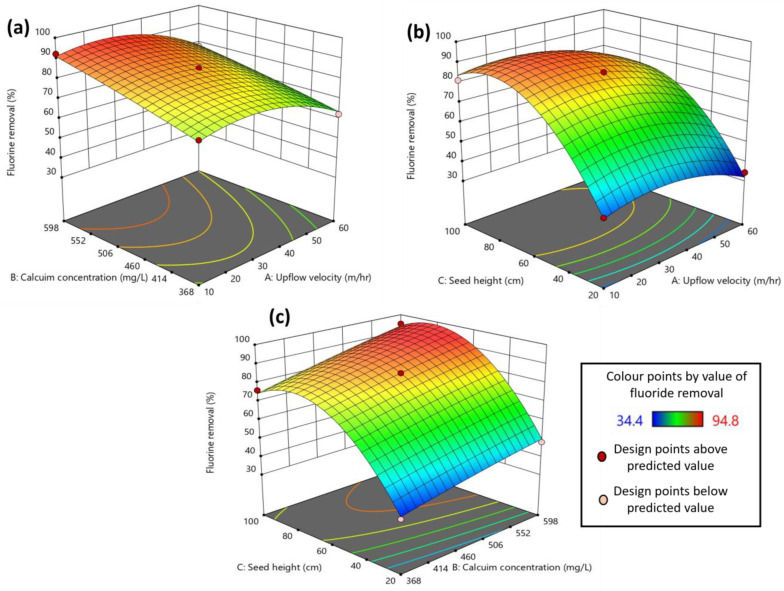
Response surface (contour) plots showing the interaction effect between process parameters on fluoride removal: (**a**) upflow velocity and calcium concentration; (**b**) upflow velocity and seed bed height; (**c**) calcium concentration and seed bed height.

The contour or surface plots between the variables on fluoride removal are depicted in Figure 4. The figure provides more insight into the interaction effect of the parameters on fluoride removal. The intense orange color in the contour plot represents the peak value of the response. An increase in the calcium concentration has a significant positive impact on the fluoride removal, as can be seen in Figure 4a,c. Calcium dosage is one of the most important parameters as it not only impacts fluoride removal by reacting with it but also significantly impacts the crystal formation. Damtie et al. [6] suggested that at high calcium concentrations, the fluoride removal was high, and the resulting crystals were of fluorite (CaF_2_) rather than kogarkoite (Na_3_(SO_4_)F), which formed at low Ca^2+^ concentration. A similar positive impact was also observed for seed bed height, with an increase in bed height increasing fluoride removal (Figure 4b,c). The reason behind such observation is that the bed height is associated with the amount of seed material available in the reactor, and that, in turn, is directly proportional to the available surface area of the particles. Hence, with an increase in the seed bed height, the surface area, i.e., the reaction area, increases, consequently increasing the pollutant handling capacity of the FBR [31]. In the case of upflow velocity, the lower range (10–20 m/h) favored better fluoride removal (Figure 4a,b). This is because, at higher upflow velocity, the fines formed inside the reactor escape from the reactor, causing an increase in fluoride concentration in the effluent [7]. Also, upflow velocity is inversely proportional to the empty bed residence time, which is the reaction time available for the reactants to adhere to the seed particles [32]. Hence, at an elevated upflow velocity, this contact time reduces significantly enough to hinder effective fluoride removal in the FBR system.

### 2.3. Characterization of Calcium Fluoride Crystals

**Figure 5:**
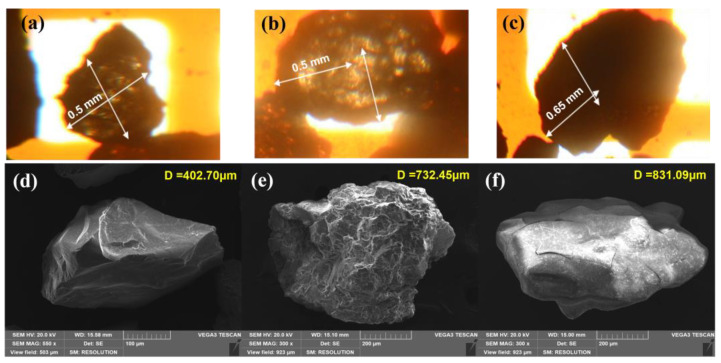
Phase contrast (**a**–**c**) and SEM microscopy (**d**–**f**) imaging of calcium fluoride crystals. (**a**,**d**) initial silica seed; (**b**,**e**) CaF_2_ crystals after 5 h; (**c**,**f**) CaF_2_ crystals after 40 d.

The calcium fluoride crystals formed in the FBR were characterized using different instrumental techniques. Figure 5a–c shows an image of CaF_2_ crystals with different reaction time periods using phase contrast microscopy. The crystal size increased by more than double from 0.4–0.5 mm diameter on 1st day to 1.0 mm diameter particle on the 40th day. Further, the outer crystal layer appears to be dense with dark color due to calcium fluoride deposition. This increase in the crystal size confirms that the deposition of CaF_2_ onto the seed materials has taken place over time. The surface of the seed crystals has provided a stable template where atoms, molecules or ions can become adsorbed, increasing the chance of forming stable bonds with each other during crystallization [33,34]. The SEM image shows the surface of the original seed (Figure 5d) and after crystal formation (Figure 5e–f). The change in the surface from rough at 5 h to a smoother surface after 40 d of an experiment can also be observed from the SEM images. A similar shape of CaF_2_ crystal formed during fluidized bed crystallization has been previously reported [33,34]. The corresponding elemental compositions of the crystal surface are provided by EDX spectra of the initial silica seed and calcium fluoride crystals (Table 3). The initial seed contained Si, O and C, which, when exposed to fluoride and calcium inside the FBR, showed peaks for the presence of other elements, including F, Ca, Na, Al, Cl and S, in addition to the original elements Si, C and O. The F and Ca composition increased from 7 and 3% (wt) in earlier samples (collected at 5 h) to 43 and 27% in the later sample (collected on 40th d). This indicates the deposition of CaF_2_ onto the seed. Furthermore, the presence of Si was not detected in the 40th d sample, which could be due to the fact that the entire seed surface is covered with deposited CaF_2_ by such a long reactor operation.

**Figure 6:**
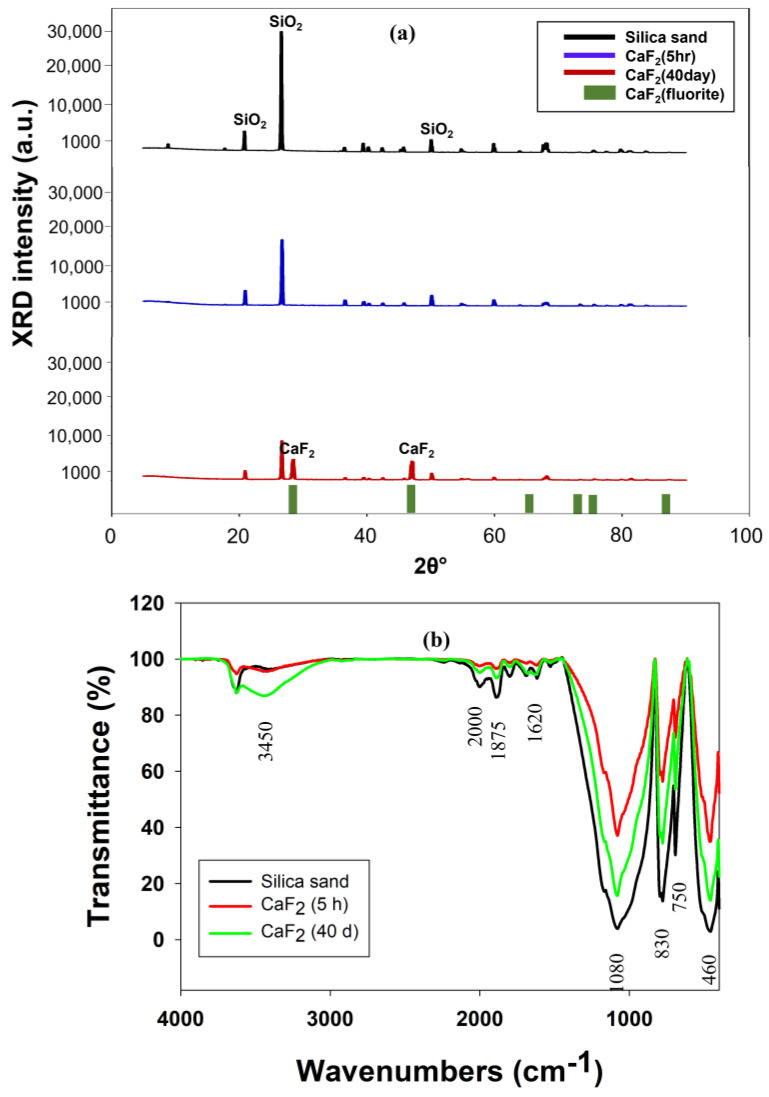
(**a**) XRD and (**b**) FTIR spectra of calcium fluoride crystals formed in FBR.

The XRD spectra of the calcium fluoride crystals formed inside the FBR are shown in Figure 6a. The original silica seed shown peaks at 2θ = 20.39°, 26.07° and 49.02°, which are typical to the SiO_2_ [18]. The sample obtained after 5 h of FBR operation was more or less similar to the SiO_2_ as the peaks present in the diffractogram matched more with SiO_2_ (Figure 6a). The amount of CaF_2_ deposited onto the seed materials after 5 h is proportionally much smaller than silica, which is the reason behind detecting SiO_2_ in this sample. However, the peak intensity for SiO_2_ in this sample was reduced compared with the original seed sample, indicating that the CaF_2_ crystallization process has started and that there are other elements in the sample along with SiO_2_. The characteristic peaks corresponding to the CaF_2_ were observed in the crystal samples obtained after 40 d reactor operation. The peaks corresponding to CaF_2_ that are present in this sample were at 2θ = 28° and 46.6° (JCPDS no. 87-0971) [35]. The formation of CaF_2_ with the FBR was confirmed with this XRD analysis. The XRD characterization of the CaF_2_ crystals also revealed the absence of calcium carbonate (CaCO_3_), calcium sulfate (CaSO_4_) or fluorapatite (Ca_5_(PO_4_)_3_F) in the resulting crystals. These compounds are known to precipitate during fluoride removal through fluidized bed crystallization and, even in some cases, are the main product instead of CaF_2_ [22,32]. The possibility of any of these compounds’ formations during FBR operation was completely eliminated through the XRD analysis, which also, in turn, highlighted the purity of CaF_2_ formed.

The FTIR spectra obtained for silica seed material and calcium fluoride crystals obtained from different time intervals are shown in Figure 6b. Table 4 provides detailed information on different peaks identified on silica seed and calcium fluoride crystals. The change in a certain peak intensity following exposure to fluoride indicates the role of this active group in the interaction with this fluoride. For example, the intensities of the −OH group at both 3450 cm^−1^ and 1620 cm^−1^ reduced, indicating the substitution of the hydroxyl group by F^−^. A similar observation was also previously made by other authors [23,36], who indicated that this replacement of the −OH group with F^−^ ion is more favorable as fluoride ion is smaller in size than −OH ion, which is better for stable crystal structure. Although it is difficult to confirm the presence of CaF_2_ with FTIR spectra alone as it can only indicate the presence of functional groups, some of the previous studies [23,36] have suggested that peak near 750 cm^−1^ is due to CaF_2_ presence, which can also be observed in this current study.

Moreover, the compositional analysis of the silica seed and CaF_2_ crystals during different time intervals was carried out using ICP-OES, and the results are shown in Table S1. For this analysis, the samples are first acid-digested, and only the acid-soluble parts are analyzed. The acid-insoluble parts of the samples were much larger than any other constituents, with 94.46% and 70% for silica seed and CaF_2_ crystal at 5 h and after 40 d, respectively. This insoluble part of the samples is silica seed used for crystal formation. The 24.5% reduction in insoluble silica parts in the 40 d sample is due to the deposition of CaF_2_ onto the seed. It can also be observed that the calcium and fluoride composition increased significantly from 0.34 and 1.53% in seed material to 9.8 and 14.71% in 40 d samples. This increase in Ca and F composition in the samples directly corresponds to the reduction in silica composition and confirms the calcium fluoride crystal formation onto the silica seed. Other elements such as sulfur, aluminum, iron, potassium, magnesium, sodium and phosphate are also present in the samples in minute quantities, which may have been contributed by either the silica seed material or by the simulated wastewater used in the study. The purity of the CaF_2_ crystals was also calculated using this study by dividing the Ca and F fractions by subtracting the number of impurities present in the crystal and then converting the resultant value to a percentage. By using this method, the purity of the CaF_2_ crystals obtained after 40 d of FBR operation is 89.37%, which is relatively higher than many other previous studies. Further increase in this CaF_2_ deposition could be achieved by a longer duration of FBR operation, which could improve the purity of the resulting crystals to a much greater value.

### 2.4. Practical Implications and Future Perspectives

In recent years, an important paradigm shift in wastewater treatment has taken place where, in order to advance towards a circular economy along with treating wastewater, it is also being considered as a source of valuable resources [41,42]. In this approach, following removal from wastewater, resources can be retrieved and recycled back into the economy, reducing the energy and emissions of different products along their life cycles and generating enough income to cover the cost of treating the water. In this context, the treatment of the fluoride-containing semiconductor industry wastewater needs to be considered. The semiconductor industry is one of the largest consumers of fresh water and consequently generates a relevantly large amount of wastewater that needs treatment prior to its release or reuse [43]. One of the major pollutants, fluoride, contained in semiconductor industry wastewater, can be successfully removed using the fluidized bed crystallization method and recovered as CaF_2_ crystals.

The crystallization mechanism involves the growth of calcium fluoride crystals on the surface of seed crystals in the metastable supersaturated zone, which lies between the solubility and supersolubility curves of calcium fluoride [33,44]. Notably, this mechanism allows calcium fluoride to precipitate as crystals without the spontaneous generation of microcrystals, making it unique in comparison to conventional coagulation/precipitation methods [7,27]. The calcium fluoride crystallization method generates minimal inorganic sludge, making it distinct from coagulation/precipitation methods, and produces high-purity calcium fluoride crystals. Due to this relatively low moisture content and high purity in the crystals than in the sludge produced by coagulation/flocculation, CaF_2_ crystals have a high degree of application potential [45]. For example, the steel industry uses large quantities of CaF_2_, which is used to reduce the melting temperature and remove impurities [33]. Often, such procurement involves high costs and the involvement of other countries. For instance, the entirety of CaF_2_ required by steel industries in South Korea depends upon foreign imports despite having a well-developed semiconductor industry. In recent times, some efforts have been put into place to integrate these two industries to use calcium fluoride-containing sludge for steel plants. However, CaF_2_ recovered in the form of crystals (rather than as sludge) could be proven much better in terms of handling and application potential in this industry.

The challenges associated with this process are mainly related to reactor operation and concern the purity of CaF_2_ crystals formed. Notwithstanding the huge application of fluidized bed reactors in wastewater treatment, their operation mostly relies on heuristics, i.e., human acumen, rather than established operational parameters [7,46]. Moreover, the CaF_2_ crystallization process also depends on many factors. In this study, all these important parameters were optimized first individually, and then their combined effect was understood through experimental design. The results from these optimization processes were further utilized to demonstrate the process efficiency of the FBR system.

Regarding purity, although a fraction of the crystal formed in this study is silica due to the use of silica as seed material in the FBR, nearly 90% purity of the CaF_2_ crystals showed their suitability to be used in industrial processes, including steel manufacturing processes. This practice of using seed materials is not unusual, and many previous studies have used such a secondary nucleation process as it provides advantages such as a faster process and low release of fines. In this study, the amount of fines produced were miniscule compared to the amount of fluoride used for crystal formation. In order to further improve the purity of the crystals, one possible strategy could be using CaF_2_ (as fines recovered from the fluoride removal process) itself as seed material [7]; future research should be focused in this direction. In addition, there are many studies involving the genetic algorithm (GA)-based method for modeling experimental data, which optimizes process parameters [47,48,49,50]. Such GA-based models are similar to the natural selection process that mimics biological evolution and are capable of solving both constrained and unconstrained optimization problems, making them ideal for these types of experiments. Hence, future objectives should also include modeling the experimental data obtained for fluoride removal and calcium fluoride crystallization efficiency in the fluidized bed reactor (FBR) using a genetic algorithm optimized artificial neural network to validate.

## 3. Materials and Methods

### 3.1. Materials and Reagents

In this study, the synthetic wastewater used was formulated to replicate the typical composition of semiconductor hydrofluoric acid wastewater [20]. The synthetic wastewater was prepared with the following characteristics: pH: 2.2; conductivity: 5850 µs/cm; F^−^: 460 mg/L; NH^4+^-N: 11.4 mg/L; SO_4_^2−^: 280 mg/L; total phosphorus (TP): 2.5 mg/L; SiO_2_: 10.3 mg/L; Al^3+^: 0.70 mg/L; Fe^2+^: 21.7 mg/L; and Mg^2+^: 0.1 mg/L.

The fluoride and calcium sources used in this study were hydrofluoric acid (55%, Hoimyung waterzen, Suncheon, Republic of Korea) and calcium chloride (35%, Hoimyung waterzen, Republic of Korea). Trace elements include glucose (≥98.5%, MB Cell, Republic of Korea), ammonium hydroxide (28.2%, Mallinckrodt, Hazelwood, MO, USA), sodium chloride (99%, Samchun, Republic of Korea), silicon dioxide (100%, Daejung, Republic of Korea) and tungsten powder (99.9%, Daejung, Republic of Korea). For seeds, two domestic types and one Australian type of silica sand were used, and two domestic types of AFM were used. The main component of silica sand and AFM material is SiO_2_. The pH was adjusted with 1 M NaOH (95%, Samchun, Republic of Korea) and 1 M H_2_SO_4_ (95%, Samchun, Republic of Korea).

### 3.2. Experimental Reactor Setup

**Figure 7:**
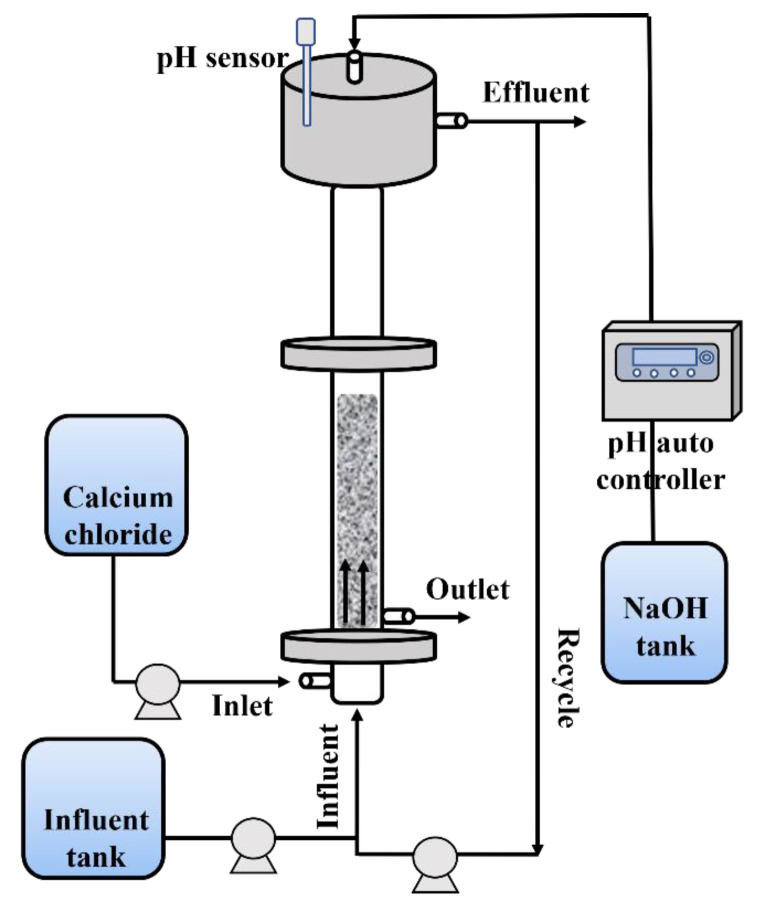
Schematic diagram of fluidized bed reactor.

In this research, a custom-built FBR system was employed for the purposes of fluoride removal and calcium fluoride crystallization. The schematic of the FBR is provided in Figure 7. The reactor was fabricated using acrylic material to allow for visual observation of the internal conditions. The FBR had an outer diameter of 50 mm and stood at a height of 2150 mm. The outlet for treated water discharge was situated approximately 150 mm below the upper wear, facilitating natural drainage. For the injection of the CaCl_2_ reagent, an inlet was positioned 400 mm above the reactor’s base. Additionally, the outlet for CaF_2_ crystal discharge was strategically placed above the point of reagent injection, ensuring optimal conditions for the formation of calcium fluoride crystals. The reactor was operated in an upward flow mode, enabling the seed particles, classified to a diameter range of 0.3 to 0.7 mm using sieves, to move within the lower section of the reactor as synthetic wastewater was introduced. To mitigate pH fluctuations arising from chemical reactions, a pH auto controller was employed to monitor and regulate the pH levels as required.

### 3.3. Lab Scale Batch Experiment

The batch experiments were conducted in a 3.44 L volume FBR filled with silica sand seed particles of diameters ranging from 0.3 to 0.7 mm (50 cm seed bed height). To achieve fluidization of the seed bed, a peristaltic pump was utilized to circulate the synthetic wastewater at a flow rate of 1.02 L/min. The experiments were carried out under ambient temperature (25 °C) and atmospheric pressure conditions. The CaCl_2_ at 35% concentration was used as a reactant, and based on the desired molar ratio of [Ca^2+^]/[F^−^], this solution was injected using a peristaltic pump for 300 min in conjunction with the flow rate of the circulating water.

Various experimental factors were considered to determine the optimal conditions for fluoride ion removal and calcium fluoride crystallization efficiency. These factors included hydraulic retention time (HRT), pH, the [Ca^2+^]/[F^−^] (mol/mol) ratio, seed type, seed size, seed bed height and upflow velocity. The detailed experimental conditions are presented in Table 5. For the batch experiments, the pH and HRT were varied in the range of 3 to 9 and 3 to 8 h, respectively. The effect of temperature was studied by taking three temperatures, namely 10, 25 and 40 °C. The calcium-to-fluoride molar ratio varied between 0.3 and 0.8. The effect of different types of seeds and their sizes on fluoride removal and calcium fluoride crystallization was studied with the following types of seeds, namely silica sand (0.3–0.7 mm), silica sand (0.7–1.2 mm) and Australian silica sand (0.3–0.7 mm). To study the impact of seed bed height, the reactor was filled at different heights such as 20, 30, 40, 50, 70 and 100 cm. The wastewater flow rates were adjusted to 512, 563, 572, 580, 596 and 614 mL/min, respectively, to achieve a fixed upflow velocity of 20 m/h for different seed bed heights. Finally, the effect of influent fluoride concentration on fluoride removal was determined by varying the fluoride concentration from 50 mg/L to 30,000 mg/L. The calcium-to-fluoride ratio was kept fixed at 0.5 in this experiment, for which the concentration of influent calcium taken was 52.5–31,500 mg/L.

The fluoride removal was calculated according to the following equation:

(6) $$Fluoride\;removal\;\left(\%\right)=\frac{{F^{-}}_{in}-{F^{-}}_{ef}}{{F^{-}}_{in}}\times 100$$

where F^−^*_in_* and F^−^*_ef_* are the influent and effluent fluoride concentration from FBR, respectively.

The crystallization efficiency was calculated using the following equation:

(7) $$Crystaillization\;efficiency\;\left(\%\right)=(1-\frac{Total\;F^{-}\;removal-F^{-}\;removal\;in\;FBR\;\;}{Total\;F^{-}\;removal}\;)\times 100\;$$

where Total F^−^ removal: F^−^ removal through crystallization (in FBR) and filtration, and F^−^ removal in FBR: F^−^ removal only through crystallization.

### 3.4. Optimization of Process Parameters Using Response Surface Methodology

Following the previous experiment to understand the effect of individual parameters on fluoride removal and calcium fluoride crystallization, three important parameters are optimized using response surface methodology (RSM). The selected parameters for this experimental design were upflow velocity (10–60 m/h), calcium concentration (368–598 mg/L) and seed bed height (20–100 cm). Table 1 presents the combination level of the parameters in each experimental run, as per the experimental design. Each of the parameters in this experiment was evaluated at three different levels, with a total of 17 experiments (Table 1). The fluoride removal was taken as a response in each individual experiment.

### 3.5. Characterization of Calcium Fluoride Crystals Formed in FBR

In order to characterize calcium fluoride crystals, a long-term continuous FBR operation was performed with all the optimized process parameters for a duration of 40 days. Following this experiment, the CaF_2_ crystals formed inside the FBR were characterized using different instrumental techniques. The SEM-EDX analysis was carried out using a single calcium fluoride crystal retrieved from the FBR. The crystal was fixed onto a metal grid with the help of an adhesive carbon tape and observed under SEM-EDX (Model Vega3, Tescan, Brno, Czech Republic). The structural composition and crystallinity of the crystalline products were measured using a high-resolution X-ray diffractometer (HR-XRD, Model D8 advance, Bruker, Billerica, MA, USA), and scans were performed within the range of 5° to 90°. A Fourier transform infrared spectrometer (FT-IR, Model Spectrum 3, Perkin Elmer, Waltham, MA, USA) was used to determine the functional groups of the crystal structure. Compositional analysis of the CaF_2_ crystal is carried out with an inductively coupled plasma optical emission spectrometry (ICP-OES Model G8014AA, Agilent, Santa Clara, CA, USA).

### 3.6. Analytical Methods

Analyses of ionic components, including fluoride, calcium and sulfate, were performed using an ion chromatograph (IC, Model Ion chromatography–mass spectrometer, Metrohm). All samples were filtered using a 0.45 μm GFC filter prior to their analysis. pH was measured using a pH meter (Model ST300, Ohaus, Parsippany, NJ, USA).

## 4. Conclusions

The fluoride-containing simulated semiconductor industry wastewater was treated using a calcium fluoride crystallization process using a fluidized bed reactor with silica seed material. The effect of process parameters on fluoride removal was studied under batch mode of operation where, at optimized conditions, an 82.5% fluoride removal along with a crystallization efficiency of 95.1% could be obtained. The fluoride removal could be further improved to 94.8% using response surface methodology, which showed that a high influent calcium concentration (598 mg/L), a high seed bed height (100 cm) and a moderate upflow velocity (35 m/h) is favorable to the process performance. The characterization of the final product obtained from FBR using SEM-EDX, HR-XRD, FTIR and ICP-OES confirmed the crystals to be of high purity CaF_2_. Overall, the findings from this study offer a fresh perspective for the advancement of environmentally friendly, high-performing technology for the innovative treatment of fluoride-containing wastewater and potential resource recovery.

## Figures and Tables

**Table 1:** RSM experimental design showing the combinations of the variables and their levels in each experimental run along with the responses.

Exp. Run	A: Upflow Velocity (m/h)	B: Calcium Concentration (mg/L)	C: Seed Bed Height (cm)	Response: Fluoride Removal (%)	RSM Predicted Values
1	60	483	100	69.3	72.7
2	10	483	20	40.3	40.3
3	35	483	60	85.2	86.9
4	35	598	20	48.1	51.9
5	35	483	60	85.2	86.9
6	10	368	60	73.9	73.6
7	35	598	100	94.8	95.7
8	60	598	60	78.0	82.4
9	10	483	100	81.2	84.2
10	60	368	60	62.5	65.9
11	35	368	100	76.1	75.7
12	60	483	20	34.4	34.9
13	10	598	60	92.2	91.6
14	35	483	60	85.2	86.9
15	35	368	20	35.1	37.6
16	35	483	60	85.2	86.9
17	35	483	60	85.2	86.9

**Table 2:** Analysis of variance (ANOVA) of fluoride removal efficiency by calcium fluoride crystallization during process parameter optimization using RSM.

Source	Sum of Squares	Degree of Freedom	Mean Square	F-Value	*p*-Value Prob > F	Remarks
Model	6354.68	9	706.08	191.14	<0.0001	Significant
A	235.44	1	235.44	63.74	<0.0001	Significant
B	536.28	1	536.28	145.18	<0.0001	Significant
C	3341.53	1	3341.53	904.60	<0.0001	Significant
AB	1.96	1	1.96	0.5306	0.4900	Not significant
AC	9.00	1	9.00	2.44	0.1625	Not significant
BC	8.12	1	8.12	2.20	0.1817	Not significant
A^2^	261.95	1	261.95	70.91	<0.0001	Significant
B^2^	1.85	1	1.85	0.5003	0.5022	Not significant
C^2^	1859.05	1	1859.05	503.27	<0.0001	Significant
Residual	25.86	7	3.69			
Lack of Fit	25.86	3	8.62			
Pure Error	0.0000	4	0.0000			
Cor Total	6380.54	16				

A: Upflow velocity; B: Calcium concentration; C: Seed bed height. Standard deviation: 1.92; mean: 71.29; C.V. %: 2.70; R^2^: 0.9959; adjusted R^2^: 0.9907; predicted R^2^: 0.9352; adequate precision: 41.8143.

**Table 3:** Elemental composition of silica seed and CaF_2_ crystals obtained after 5 h and 40 d using EDS analysis.

Element	At. No.	Silica Seed		CaF_2_ Crystal (5 h)		CaF_2_ Crystal (40 d)	
		Weight (%)	Atom (%)	Weight (%)	Atom (%)	Weight (%)	Atom (%)
C	6	11.32	16.93	8.84	13.71	10.49	17.60
O	8	54.61	61.30	48.13	56.02	16.61	20.61
F	9	ND	ND	6.84	6.71	43.08	45.68
Na	11	ND	ND	0.48	0.39	1.81	1.59
Al	13	ND	ND	0.33	0.23	ND	ND
Si	14	34.07	21.78	32.77	21.73	ND	ND
S	16	ND	ND	ND	ND	1.10	0.69
Cl	17	ND	ND	0.04	0.02	0.11	0.06
Ca	20	ND	ND	2.56	1.19	26.79	13.47

ND: Not detected.

**Table 4:** FTIR peaks assignment of silica seed and calcium fluoride crystal formed in the FBR.

Wavenumbers (cm^−1^)	Functional Group	Reference
3450	−OH stretching vibrations	[23,37]
2000	C=C stretching vibrations	[36]
1875	C=O stretching vibrations	[36]
1620	−OH bending vibrations	[23]
1080	Asymmetric stretching vibrations of Si–O–Si bond	[38]
830	C=C bending vibration	[39]
750 and 460	Presence of sulfate and phosphate groups	[23]
460	Si–O stretching vibration	[40]

**Table 5:** Experimental conditions for batch optimization test.

Parameter	Values
HRT (h)	3, 4, 5, 6, 7, 8
pH	3, 4, 5, 5.5, 6, 7, 8, 9
[Ca^2+^]/[F^−^] (mol/mol) ratio	0.3, 0.4, 0.5, 0.6, 0.7, 0.8
Upflow velocity (m/h)	10, 20, 30, 40, 50, 60
Seed size (mm)	Silica sand (0.3–0.7), silica sand (0.7–1.2), silica sand Australia (0.3–0.7)
Seed bed height (cm)	20, 30, 40, 50, 70, 100
Fluoride concentration (mg/L)	50, 100, 300, 500, 1000, 5000, 10,000, 20,000, 30,000

## Data Availability

Data is contained within the article.

## Supplementary Materials

The following supporting information can be downloaded at: https://www.mdpi.com/article/10.3390/ijms25073960/s1.
