# Supplementary material for: Optimization of Calcium Fluoride Crystallization Process for Treatment of High-Concentration Fluoride-Containing Semiconductor Industry Wastewater
Source: Int J Mol Sci. 2024 Apr 2;25(7):3960. doi: 10.3390/ijms25073960 (PMC11011877; doi:10.3390/ijms25073960)
Supplement: Supplementary file 1 [file ijms-25-03960-s001.zip › ijms-2947272-supplementary.pdf]

# Optimization of Calcium Fluoride Crystallization Process for Treatment of High-Concentration Fluoride-Containing Semiconductor Industry Wastewater

**Arindam Sinharoy, Ga-Young Lee and Chong-Min Chung ***

Department of Environmental Science & Biotechnology, Jeonju University, Jeonju 55069, Republic of Korea

* Correspondence: cmchung@jj.ac.kr; Tel.: +82-632202286

## Supplementary data

**Table S1.** Composition of silica seed and $CaF_2$ crystals at different time interval using ICP-OES analysis.

| Elements | Silica seed | $CaF_2$ – 5 h | $CaF_2$ – 40 d |
|---|---|---|---|
| Sulfate | 0.14 | 0.24 | 1.19 |
| Aluminum | 0.34 | 0.23 | 0.42 |
| Calcium | 0.34 | 0.28 | 9.8 |
| Fluoride | 1.53 | 1.31 | 14.71 |
| Copper | 0.01 | 0.01 | 0 |
| Iron oxide | 0.7 | 0.45 | 0.57 |
| Potassium | 0.27 | 0.33 | 0.2 |
| Magnesium | 0.12 | 0.08 | 0.16 |
| Sodium | 0.38 | 0.00 | 0.52 |
| Phosphate | 0.13 | 0.31 | 0.04 |
| Zinc | 0.04 | 0.09 | 0.01 |
| Silica (acid soluble) | 0.35 | 0.30 | 1.18 |
| Acid insoluble | 94.46 | 95.27 | 70 |
